# Natural history of cervical intraepithelial neoplasia in pregnancy: postpartum histo-pathologic outcome and review of the literature

**DOI:** 10.1186/s12884-016-0861-8

**Published:** 2016-04-07

**Authors:** Mariella Mailath-Pokorny, Richard Schwameis, Christoph Grimm, Alexander Reinthaller, Stephan Polterauer

**Affiliations:** Department of Obstetrics and Gynecology, Division of Obstetrics and Feto-maternal Medicine, Medical University of Vienna, Vienna, Austria; Gynecologic Cancer Unit, Comprehensive Cancer Center, Department of Obstetrics and Gynecology, Division of General Gynecology and Gynecologic Oncology, Medical University of Vienna, Vienna, Austria; Karl Landsteiner Institute for General Gynecology and Experimental Gynecologic Oncology, Vienna, Austria

**Keywords:** Cervical intraepithelial lesion, Pregnancy, Regression, Persistence, Regression, HPV

## Abstract

**Background:**

To study the natural history of cervical intraepithelial neoplasia (CIN) during pregnancy and to compare the rates of persistence, progression and regression of CIN by colposcopically guided biopsy (CGB) during pregnancy with outcome in non-pregnant-women.

**Methods:**

A retrospective analysis of all pregnant women diagnosed with CIN at our outpatient clinic between 2005 and 2010 was performed. A CGB for histo-pathological analysis was obtained in all participants and observational management was performed. The histo-pathologic findings of initial and postpartum visits were collected. Rates of persistence, progression and regression of CIN were assessed. Results were compared to a matched control group of non-pregnant women where observational management was performed for at least three months. In addition a review of the literature and pooled analysis of published data was performed.

**Results:**

A total of 51 pregnant women with CIN were included into analysis. CIN 1, 2, and 3 was diagnosed by CGB in 33.3, 13.7 and 52.9 % of all pregnant women, respectively. The postpartum histo-pathologic evaluation of the pregnant cohort revealed a significantly higher tendency to spontaneous regression (56.9 versus 31.4 %, *p* = 0.010) and a considerably, but not significantly higher complete remission rate (41.2 versus 27.5 %, *p* = 0.144) when compared to the non-pregnant cohort. In addition, we observed a significantly lower CIN persistence rate than in the non-pregnant cohort (39.2 versus 58.8 %, *p* = 0.048). The progression rate was notably low in the pregnant cohort (3.9 %) and no progression to invasive cancer was observed.

**Conclusions:**

CIN lesions show considerably high spontaneous regression rates postpartum. Once presence of invasive cancer is ruled out definitive treatment can be deferred to the postpartum period.

## Background

The prevalence of cervical intraepithelial neoplasia (CIN) in the population of pregnant women is approximately 1 % [1]. The recent literature recommends a cervical cytology-based screening as part of routine prenatal care, since most of the precursor lesions of cervical cancer occur in young women within the child bearing age [2]. Moreover, the incidence of persistent HPV infection, which is associated with the development of CIN is highest in women aged 20–25 years [3–6]. The trend of managing intraepithelial abnormalities during pregnancy has changed over the years from an aggressive surgical management to a more conservative approach. Once invasive disease has been ruled out, observational management with regular gynecological examinations including PAP-smear, colposcopy and colposcopy-guided biopsy (CGB) has been proven to be safe and is recommended by expert groups [7–10]. However the published literature reveals heterogeneous data about regression rates in pregnant women with CIN I between 32 and 69 % and CIN II-III between 16.7 to almost 70 %, respectively [7, 9, 11–14]. Additionally persistence rates, ranging from 38.4 up to 70 % [8, 16, 17] have been reported, thus leading to the recommendation of a close postpartum follow-up [8, 11, 16, 18].

The aim of the present report was to study the natural history of histo-pathologically confirmed CIN lesions in pregnant women and to assess postpartum rates of regression, persistence, and progression. We used a robust data set obtained at a large, single center with a close follow-up of patients. In addition, a review of the current literature and pooled data analysis was performed.

## Methods

All consecutive patients, that were initially referred to the institution’s outpatient clinic due to an abnormal PAP test result and had a diagnostic work up including cervical punch biopsy, were documented. Data of all women, who were diagnosed with CIN between January 1, 2005 and December 31, 2010, were collected and analyzed. All pregnant women were identified and included into this study. A control group of non-pregnant women matched for CIN grade was selected. Women of both groups had observational management for at least 3 months and were diagnosed within the same observation period. The institution’s electronic documentation system was used for data collection and patients’ charts were analyzed to assess demographic and clinical-pathological data. In the course of routine diagnostic work-up at the patients’ first visit at the institution a questionnaire was used to inquire patients’ medical history and demographic data. Initial diagnostic work-up for patients with abnormal PAP test results included the following: colposcopy, repeated PAP smears, and CGB of all suspicious lesions. Women with pathological cytology and negative or equivocal colposcopy were biopsied from all four quadrants of the uterine cervix. Human papilloma virus (HPV) testing using the Hybrid Capture® HPV DNA test was performed based on the physician’s decision. In pregnant women clinical examinations including PAP smear and colposcopy with or without CGB were performed in each pregnancy trimester in order to rule out presence of invasive disease. Postpartum visits were scheduled approximately eight weeks after delivery and CIN status was assessed using CGB. Non-pregnant women with CIN were seen every 3–6 months and examined with colposcopy and CGB. We assessed CIN persistence, regression, and progression rates within the observational time period. Results of cervical punch biopsies were used for the assessment of natural history of CIN and CIN status in pregnant and non-pregnant women. Persistence, regression, and progression rates at last follow-up visit were assessed based on histologic findings from biopsy or conization specimens. Regression at a subsequent visit was defined as a lower grade lesion than that found at the initial visit. Persistent disease was defined as CIN of same grade as found at the initial diagnostic work up. Disease progression was defined as histologic evidence of higher CIN grade at subsequent visit compared to the initial visit. HPV clearance was assessed, if HPV status was available at the beginning and the end of the observation period.

We used descriptive statistics for visualization of patients’ demographic data. Values are given as mean (standard deviation [SD]) when normally distributed or as median (range) at presence of skewed distribution. *P*-values of <0.05 were considered statistically significant. We used the statistical software SPSS 21.0 for Windows (SPSS 21.0, SPSS Inc, Chicago, IL, USA) for statistical analyses. The impact of pregnancy on qualitative outcome variables (i.e., regression vs. no regression, complete regression vs. no complete regression, persistence vs. no persistence and progression vs. no progression) was assessed by using the Chi-squared test. Binary logistic regression analysis was performed to assess odds ratios and 95 % confidence intervals (95 % CI) as well as to adjust for covariables like histological grade, age, and smoking status (Table 2). The impact of histological grade of pregnant women at time of diagnosis on qualitative outcome variables (i.e., regression vs. no regression, complete regression vs. no complete regression, persistence vs. no persistence and progression vs. no progression) was assessed. Binary logistic regression analysis was performed to assess odds ratios and 95 % confidence intervals (95 % CI) (Table 3). In January 2014, a Pubmed search using the terms “cervical intraepithelial neoplasia” or “CIN” or “cervical dysplasia” and “pregnancy” or “pregnant women” was performed to identify articles on this topic published in the English literature. Only studies reporting histo-pathological postpartum outcome were selected and reviewed. A pooled analysis of regression, persistence, and progression rates were performed. For Table 4 data from published articles were assessed and combined in the pooled analysis, a formal meta-analysis was not performed.

Study approval was obtained from the institutional review board (IRB) of the Medical University of Vienna (ECS 1213–2011). Due to the retrospective design of the present study the institution’s IRB granted a waiver of consent. Therefore no informed consent was obtained.

## Results

Within the study period 51 pregnant women with histologically proven CIN were identified and included into the analysis. We observed CIN I in 17 (33.3 %), CIN II in 7 (13.7 %) and CIN III in 27 (52.9 %) pregnant women, respectively. Mean (standard deviation (SD)) gestational age at diagnosis was 15.0 (6.3) weeks. During the same period of diagnosis we identified a control group of 51 consecutive non-pregnant women, which was matched by CIN grade at diagnosis in order to compare the natural history of CIN in pregnant versus non-pregnant women. Characteristics of pregnant and non-pregnant women with CIN are shown in Table 1. Parity was significantly higher in the group of pregnant patients. No statistically significant difference was found between the cohorts with respect to HPV status, age and smoking history. Mean (SD) follow-up time in pregnant women and non-pregnant women was 36.4 (29.0) and 51.6 (30.5) weeks, respectively. The main difference in observation time was observed in pregnant and non-pregnant women with CIN 3 (*p* = 0.003). Mean (SD) observation time was 20.4 (19.5) weeks and 47.5 (30.1) weeks in non-pregnant and pregnant women with CIN 3, respectively (*p* < 0.001). In Table 2 the rates of persistence, progression, regression, and complete remission of CIN diagnosed by CGB at follow-up examination for pregnant and non-pregnant women are provided. Adjusted and unadjusted odds ratios and 95 % confidence intervals are shown. Pregnant women showed significantly higher spontaneous regression rates. For pregnant and non-pregnant women HPV clearance rates (95 % CI) were 26.7 % (0.72–2.10) and 38.9 % (0.36–1.52), respectively. In addition we observed a significantly lower persistence rate than in non-pregnant controls. The progression rate was notably low in the pregnant cohort and no cases of invasive cancer were observed within the observation period. All of the 51 pregnant women had regular postpartum follow-up visit starting 8 weeks after delivery with a mean (SD) follow up time of 43.9 (30.6) weeks after delivery. In our series we observed 50 live births and one pregnancy loss. A cesarean section rate of 34.1 % was noted. Rates of histo-pathological outcome parameters of pregnant patients broken down by CIN grade within the observation period are shown in Table 3. In total, 24 (47.1 %) patients of the pregnant cohort underwent conisation at a mean (SD) time of 44 (22.8) weeks postpartum. Table 4 provides a review of the published literature and pooled analysis of studies reporting outcome rates of CIN in pregnancy where histo-pathological outcome data were available. In total 364 articles were found matching the search criteria. Only data from studies where histological baseline and outcome information was available were summarized together with the presents study within the pooled analysis (*n* = 8). Pooled analysis revealed overall regression, persistence, and progression rates of 46.8, 43.6, and 9.6 % for pregnant women with CIN, respectively [7–9, 11, 15–17, 19].

**Table 1:** Patients’ characteristics at study inclusion

Parameter	Pregnant Women	Non-pregnant Women	*p*-Value
	N (%), mean (SD)	N (%), mean (SD)	
Total	51	51	
Histological grade			(matched)
CIN 1	17 (33.0)	17 (33.0)	
CIN 2	7 (13.7)	7 (13.7)	
CIN 3	27 (52.9)	27 (52.9)	
HPV high-risk			0.645
Positive	25 (86.2)	38 (92.7)	
Negative	4 (13.8)	3 (7.3)	
Missing	21	10	
Age	30.2 (5.5)	30.6 (6.0)	0.672
Parity	1.88 (1.2)	1.03 (1.3)	0.005
Smoking Status			0.221
Smoker	21 (70.0)	14 (60.9)	
Non-smoker	9 (30.0)	9 (39.1)

**Table 2:** Rates of histo-pathological outcome parameters within the observation period

Parameter	Pregnant Women	Non-pregnant Women	*p*-Value	OR (95 % CI)	AOR (95 % CI)^a^
	N (%)	N (%)			
Total	51	51			
Persistence	20 (39.2)	30 (58.8)	0.048	0.45 (0.20–0.99)	0.21 (0.05–1.02)
Progression	2 (3.9)	5 (9.8)	0.240	0.38 (0.07–2.03)	0.28 (0.02–4.45)
Regression	29 (56.9)	16 (31.4)	0.010	2.88 (1.28–6.49)	7.02 (1.33–37.22)
Complete Remission	21 (41.2)	14 (27.5)	0.144	1.85 (0.81–4.24)	4.12 (0.54–31.43)

*OR* odds ratio, *AOR* adjusted odds ratio, 95%*CI* 95 % confidence interval

^a^adjusted for covariables: histological grade, age, and smoking status

**Table 3:** Rates of histo-pathological outcome parameters of pregnant patients broken down by CIN grade

Parameter	CIN 1	CIN 2	CIN 3	*p*-Value	OR (95 % CI)
	N (%)	N (%)	N (%)		
Total	17	7	27		
Persistence	2 (11.8)	1 (14.3)	17 (63.0)	0.002	4.01 (1.68–9.61)
Progression	1 (5.9)	1 (14.3)	0	0.32	4.02 (0.07–2.38)
Regression	14 (82.4)	5 (71.4)	10 (37.0)	0.005	0.34 (0.16–0.72)
Complete Remission	14 (82.4)	5 (71.4)	2 (7.4)	0.001	0.13 (0.05–0.33)

*OR* odds ratio, 95 % *CI* 95 % confidence interval

**Table 4:** Review of the literature and pooled analysis of studies with reported histo-pathological outcome

Author, Date	N	Population	Analysis	Regression	Persistence	Progression
Lurain [19]	53	Pregnant women with CIN I–III	Retrospective	77.4 %	22.6 %	0 %
Yost [7]	153	Pregnant women with CIN II–III	Retrospective	69.3 %	26.8 %	3.9 %
Palle [16]	142	Pregnant women with CIN I–III	Retrospective	25 %	47 %	28 %
Vlahos [8]	78	Pregnant women with CIN II–III	Retrospective	61.6 %	38.4 %	0 %
Paraskevaides [9]	64	Pregnant women with CIN II–III	Retrospective	37.5 %	59.4 %	3.1 %
Serati [15]	36	Pregnant women with CIN II–III	Prospective	47.3 %	52.7 %	0 %
Coppolillo [11]	30	Pregnant women with CIN II–III	Retrospective	16.7 %	70.0 %	13.3 %
Kärrberg [17]	163	Pregnant women with CIN I–III	Prospective	33.1 %	54.6 %	12.3 %
This study	51	Pregnant women with CIN I–III	Retrospective	56.9 %	39.2 %	3.9 %
Pooled analysis	770	Pregnant women with CIN I–III	Pooled	46.8 %	43.6 %	9.6 %

## Discussion

The present study revealed higher spontaneous regression rates and lower persistence rates for CIN I–III in pregnant women when compared to non-pregnant women. We observed a notably low rate of disease progression and no cases of progression to invasive disease during the postpartum follow-up period. Our data support the opinion that once invasive disease can be excluded by colposcopy and CGB, definitive CIN therapy in pregnant women can be deferred until after delivery. In the current guideline of the American Society of Colposcopy and Cervical Pathology (ASCCP) observational management for women diagnosed with CIN in pregnancy is recommended [10].

The incidences of CIN and HPV infection in pregnant women are comparable to that of non-pregnant women [20, 21]. The initial obstetrical consultation provides an excellent opportunity to detect patients with abnormal PAP smears [2, 20, 21]. Historically, women with high-grade CIN were treated by cone biopsy during pregnancy [22, 23, 24]. Several studies reported that cone biopsy in pregnancy is associated with an impaired pregnancy outcome [25, 26]. Other reports showed that loop electrosurgical excision procedures are safe during pregnancy with a miscarriage rate <1 % [17]. Due to the low rates of progression during pregnancy it is nowadays accepted that most patients may safely undergo expectant management if invasive disease has been ruled out [13, 27]. Within the last decades, several authors studied the natural history of CIN diagnosed during pregnancy with varying outcome (Table 4).

Our study reports a significantly higher postpartum regression rate of CIN in the group of pregnant patients than in the non-pregnant group. The high regression rate is in accordance to recent previous studies, which report regression rates between 37 and 74 % for pregnant women at the time of post-partum follow-up [7–9, 12, 14, 15, 19]. Additionally we observed a 82 % regression rate of CIN I lesions, which is even higher than the results reported by Serati et al. [15]. Nevertheless, as reflected by the 95 % CI the results need to be interpreted with caution due to the limited number of cases. Many theories to explain these high regression rates can be found in the recent literature and among them most commonly the ones discussed below. Some authors hypothesized that, in women with HPV infection, the typical hormonal pattern during pregnancy induces a viral activation that subsequently leads to increased spontaneous regression rates after delivery [15]. Other authors speculated that regression rates could theoretically be increased by the performance of multiple cervical biopsies in the antepartum evaluation, thereby giving the appearance of spontaneous regression [14]. On the other hand in the study by Ahdoot et al. the rate of regression was higher for women who had no histologic cervical biopsy as part of the antenatal evaluation compared with women who underwent a biopsy (70 versus 52 %) [14]. Alternatively other studies suggested a correlation between CIN course and mode of delivery and found a higher rate of regression of cervical dysplasia in association with vaginal delivery compared with cesarean section (67 versus 13 %) [14, 28]. A possible mechanism for this finding may be the loss of the dysplastic cervical epithelium during cervical ripening and the passage of the fetus through the birth canal [9, 29]. Other studies have not reported any differences in regression rates among patients who delivered vaginally or by cesarean section [7, 30]. In the present study, no differences between regression rates after vaginal delivery (53.6 %) and after cesarean section (66.6 %) were observed. However, due to the limited number of pregnant women when broken-down by mode of delivery we were not able to investigate the association between regressions rates and mode of delivery adequately.

In pregnant women with a diagnosis of CIN II–III the progression rate to invasive carcinoma ranges from 2 to 28 %, according to different reports. Ackermann et al. and Everson et al. have reported progression rates of 2.4 % [31] and 3 % [32], whereas Coppola et al. [30], Kaplan et al. [33], Kaerrberg et al. [17] and Coppolillo et al. [11] reported higher rates of 8, 11, 12 and 13 % respectively. Palle et al. reported the highest rate of progression to invasive carcinoma during the postpartum period with an incidence of 28 % [16]. In our study, the progression rate was notably low in the pregnant cohort. We observed an incidence of 5.9 and 14.3 % progression in the CIN I and CIN II group, respectively. No case of invasive carcinoma was diagnosed postpartum, which is consistent with the findings of other authors [7, 9, 15]. In addition we observed a significantly lower persistence rate than in non-pregnant controls (39.2 versus 58.8 %). Our persistence rate is also lower than that reported previously ranging from 47 to 89 % [11, 16, 30, 33]. This difference might be related to variations in the methods used as well as the follow-up time of the study cohort. Additionally one may argue that these inconsistent data might be related to disparities in HPV prevalence and HPV types in the different study populations. Unfortunately, due to the retrospective study design, we cannot present type-specific HPV data. Additionally a notable number of patients did not have any HPV status assessment. Previous studies have shown that CIN is more common among smokers [34]. In our study we found a notable high smoking rate of 70 % in the pregnant population, which was slightly higher compared to the 61 % smoking rate in the non-pregnant cohort. This high rate does not clearly support the theory that regression rates are lower among pregnant smokers [12], although our dataset is clearly too small to draw any conclusion. The high rate of smokers within our cohort has to be kept in mind when interpreting our findings with respect to other populations.

Limitations of our study include the retrospective study design as well as the limited number of cases. In addition the limited data on HPV status and the lack of type-specific HPV data has to be recognized as an important limitation of the present study. We did not consider cytology results in the present study. Both HPV status and cytology results might give important additional clinical information and might influence management decisions. Another limitation might be caused by potential surveillance bias. Pregnant women are potentially more likely to participate in cytology screening programs than non-pregnant women. One might argue that therefore the pregnant women group might contain more early lesions with potentially benign biologically behavior. On the other hand, a recent study compared screening results in pregnant and non-pregnant women and could not detect any difference between CIN incidence [35]. Follow-up algorithms slightly differed between pregnant and non-pregnant women. In addition follow-up times were significantly longer for pregnant women. Especially non-pregnant women with CIN3 had a significantly shorter observation period than pregnant women. These findings might have positively influenced the rate of regression in the pregnant cohort and has to be regarded as relevant limitation of our study. However, we present data from a single, tertiary referral center using a robust, prospectively obtained database with a complete follow-up of the study patients. Additionally the complete cytologic and histo-pathologic work-up was performed by one laboratory, which may rule out interobserver variablity and therefore may reflect a strength of our study. We think that our findings are clinically meaningful and can be interpreted in the light of other studies reviewed in this report.

## Conclusion

Our study suggests that a conservative management of CIN in pregnancy is safe since we report high regression rates and low progression rates after delivery. Our findings support the recommendation of the ASCCP that conservative management of CIN in pregnancy is safe. Within the postpartum period relatively high regression rates of CIN and notably low rates of disease progression were observed in our cohort.
